# Dipeptidyl peptidase-4 inhibitors have adverse effects for the proliferation of human T cells

**DOI:** 10.3164/jcbn.17-64

**Published:** 2018-04-03

**Authors:** Noriyuki Kitagawa, Masahide Hamaguchi, Saori Majima, Takuya Fukuda, Toshihiro Kimura, Yoshitaka Hashimoto, Muhei Tanaka, Masahiro Yamazaki, Naoto Nakamura, Michiaki Fukui

**Affiliations:** 1 Department of Endocrinology and Metabolism, Graduate School of Medical Science, Kyoto Prefectural University of Medicine, 465 Kajii-cho, Kawaramachi-Hirokoji, Kamigyo-ku, Kyoto 602-8566, Japan

**Keywords:** dipeptidyl peptidase-4 inhibitor, human T cell, T cell receptor signaling, immunomodulatory effect

## Abstract

Dipeptidyl peptidase-4 (DPP-4) is a critical molecule for the metabolism of incretins. In addition, DPP-4 is known as CD26, the receptor of T cells, and plays important role in activation of T cells. Recently, DPP-4 inhibitors (DPP4i) are reported to have several immunologic effects beyond glycemic control. DPP4i seem to have anti-inflammatory effects in patients with type 2 diabetes. This might be direct effects on T cells. However, the close mechanism is not clear. To evaluate the possibility, we performed *ex vivo* assays by using primarily human CD4^+^ T cells (CD4) and CD8^+^ T cells (CD8). We purified primary naïve CD4 and CD8 from human peripheral blood. Then, we evaluated the effect of DPP4i on the proliferation of naïve T cells and the cytokine production in *ex vivo* experiments. The proliferation of CD4 and CD8 were suppressed by adding DPP4i in a dose dependent manner. However, DPP4i did not inhibit cytokine production from CD4. It was revealed by phospho-flow that the T cell receptor (TCR) signaling was attenuated in the presence of DPP4i. Taken together, DPP4i modulated TCR signaling, which contributed to attenuate the proliferation of CD4 and CD8. DPP4i have adverse effects for the proliferation of human T cells.

## Introduction

Dipeptidyl peptidase-4 (DPP-4) is a critical molecule for the metabolism of incretin.^(1,2)^ DPP-4/CD26 is also present in various tissues and cells including lymphocytes and monocytes as a transmembrane glycoprotein and is associated with immunoregulatory functions.^(3–5)^ DPP-4/CD26 has an essential role in human T cell physiology, especially in response to memory antigens besides its ability to regulate the effect of biological factors through DPP-4 enzyme activity.^(5–7)^ Furthermore, CD26 mediate co-stimulation in human CD8^+^ T cells and plays a key role in acquired immune response.^(8)^

DPP-4 inhibitors (DPP4i) improve glycemic control and have been used in diabetic therapy.^(1,9,10)^ Aside from the metabolic effects, DPP4i are known to have immunological effects, even though long-term DPP4 inhibition are not fully understood. Clinically, treatment with DPP4i reduced the number of CD4^+^ T cells.^(11)^ It is also reported that patients with type 2 diabetes who initiated combination therapy with metformin plus DPP4i decreased risk for rheumatoid arthritis and other autoimmune disease compared with those who initiated combination therapy with metformin plus sulfonylureas, thiazolidinediones or meglitinides in a large population-based cohort study.^(12)^

Consequently, DPP4i have immunomodulatory functions and play an important role in the proliferation and activation of human T cells, although the close mechanism is not fully clear.

In the current study, we supposed that one of the immunomodulatory effects of DPP4i might be derived from effects via modulation of T cell receptor (TCR) signaling especially. So, we investigated how DPP4i modulated the migration of pathogenic CD4^+^ T cells and CD8^+^ T cells in human, and whether DPP4i had immunomodulatory functions.

## Materials and Methods

### Collection and preservation of peripheral blood mononuclear cells

All procedures were approved by the local Research Ethics Committee of Kyoto Prefectural University of Medicine (ERB-C-211) and were conducted in accordance with the Declaration of Helsinki, and informed consent was obtained from all.

For *ex vivo* experiments, peripheral blood (50 to 100 ml per subject) of four healthy male subjects aged 32 to 38 years was drawn by heparinized venous puncture at the forearm. Peripheral blood mononuclear cells (PBMCs) were isolated by Lymphoprep (Axis-Shield, Oslo, Norway) density gradient centrifugation and cryopreserved at −80°C using Mr. Frosty Container (Thermo Fisher Scientific, Roskilde, Denmark). PBMCs were thawed in water bath at 37°C before each experiment. PBMCs were suspended by 100 µl of Fc block (dilution; 1:1,000 in PBS/2% FBS), incubated for 10 min at room temperature and added 100 µl of surface antibody mixture (dilution; 1:100 in PBS/2% FBS) on ice, 30 min in the dark. Then, we analyzed the PBMCs by using FACS CANTO II (BD Biosciences, San Jose, CA).

### Naïve CD4^+^ and CD8^+^ T cells separation

Naïve CD4^+^ T cells were purified by an MACS separator (Miltenyi Biotec, Bergisch Gladbach, Germany), using Naïve CD4^+^ T Cell Biotin-Antibody Cocktail II and a Naïve CD4^+^ T Cell MicroBead Cocktail II and LD column according to the manufacturer’s instructions (Miltenyi Biotec). CD8^+^ T cells were purified by an MACS separator, using CD8^+^ T Cell MicroBead and LS column according to the manufacturer’s instructions (Miltenyi Biotec). The purity of naïve T cells was assessed at 96% by using FACS CANTO II.

### Proliferation assay

We aimed to investigate if DPP4i had some effects for the proliferation of effector CD4^+^ and CD8^+^ T cells. We therefore observed whether DPP4i had a harmful effect for CD4^+^ and CD8^+^ T cells. For this aim, we examined cytotoxicity or inhibition of cell proliferation in cell proliferation assay.

We performed a cell proliferation assay using the CellTrace^TM^ Violet Cell Proliferation Kits (Thermo Fisher Scientific), which is used for labeling of cells to trace multiple generations using dye dilution by flow cytometry. We cultured naive CD4^+^ and CD8^+^ T cells, which were stained by CellTrace^TM^ Violet reagent according to the manufacturer's instructions, in 200 µl of complete RPMI with anti-CD3 and anti-CD28 antibodies coated MicroBeads (2.5 µl/L × 10^5^ cells) by adding DPP4i (0, 1, 10, 20, 50 and 100 µM) in a culture plate for 5 days at 37°C, 5% CO_2_. Moreover, we used Fixable Viability Dye (FV) that can be used to irreversibly label dead cells prior to fixation and/or permeabilization procedures. Then, we studied whether DPP4i have cytotoxicity or inhabitation of cell proliferation, or measured whether the proportion of effector CD4^+^ T cells or CD8^+^ T cells was changed or not by FACS CANTO II.

### Phospho-flow analysis

We also aimed to investigate if DPP4i modulated the TCR signaling and suppressed induction of effector T cells from naïve CD4^+^ T cells. To clarify the mechanism, we investigated whether intracellular kinase subsequent to TCR signaling was changed or not by adding DPP4i in extracellular-signal-regulated kinases (ERK), p38 mitogen-activated protein kinase (MAPK) and Akt. We examined phospho-flow to check the expression levels of the phosphorylation of phospho ERK (pERK) 1/2 and phospho p38 MAPK (pp38 MAPK) which were the dominant phospho protein downstream of TCR signaling. We also examined phospho-flow to check the expression levels of the phosphorylation of phospho Akt (pAkt).

We performed phospho-flow to measure the phosphorylation of intracellular kinase according to the manufacturer’s instructions (BD Biosciences). PBMCs were stimulated by anti-CD3 antibody (1 µg of antibody per 50 µl of PBMCs, eBioscience, San Diego, CA) and anti-CD28 antibody (2 µg of antibody per 50 µl of PBMCs, eBioscience) for 15 min after allowed to rest in complete media with DPP4i (100 µM) for 2 h at 37°C, 5% CO_2_. Thereafter, samples were stained intracellularly with anti-bodies.

### Cytokine assay

After washed twice with PBS/2% FBS, for stimulating interferon-γ (IFN-γ) and tumor necrosis factor-α (TNF-α) release from T cells, PBMCs were cultured in 200 µl of complete RPMI added phorbol-12-myristate-13-acetate (PMA; 5 ng/ml), ionomycine (IONO; 500 ng/ml), BD GolgiStop (0.65 µl/ml, BD Biosciences) and DPP4i (0 to 10 µM) for 4 h at 37°C. After washed twice with PBS/2% FBS, PBMCs were resuspended in 100 µl of eBio Cytofix/CytoPerm solution (1:3) and incubated on ice for 20 min in the dark. After washed twice with eBio 10 × Perm Buffer, 100 µl of cytokine stain mixture (dilution: 1:50 in 10 × Perm Buffer) and incubated on ice, 30 min in the dark. After washed twice with eBio 10 × Perm Buffer and once with PBS/2% FBS, PBMCs were resuspended in 200 µl of PBS/2% FBS and transferred to BD Falcon-round-bottom tubes (BD Biosciences) for acquisition on a flow cytometer.

### Antibodies

The following antibodies were obtained from BD Pharmingen (San Diego, CA), eBioscience and Biolegend (San Diego, CA), and were used for the flow cytometry analysis: anti-CD4 (L200), anti-CD8 (RPA-T8), anti-ERK1/2 (20A), anti-Akt (pT308), anti-p38 MAPK (pT180/pY182), INFγ (4S.B3), TNFα (MAb11), IL2 (MQ1-17H12). CD3/CD28 Dynabeads (Invitrogen, Carlsbad, CA) were used for *in vitro* T-cell stimulation.

### Real-time reverse-transcription polymerase chain reaction

Several gene expressions were analyzed by real-time quantitative reverse-transcription polymerase chain reaction (RT-PCR) using the TaqMan system based on real-time detection of accumulated fluorescence. Total RNA was extracted from naïve CD4^+^ T cells or CD8^+^ T cells, which were incubated for two days, by RNeasy Micro Kit (Qiagen Japan, Tokyo, Japan). The cDNA was synthesized by reverse transcription with High Capacity cDNA Reverse Transcription Kits (Applied Biosystems, Foster City, CA). Quantitative real-time RT-PCR was performed using StepOnePlus (Applied Biosystems), followed by analysis involving software detection system (The StepOne Software, Applied Biosystems). VIC labeled probe for HPRT1 (Applied Biosystems, Rack ID: 186890765_2) was used for normalization. FAM labeled probe for BCL2 and BCL2l1 (Applied Biosystems, Rack ID: 186890765_2) were used for target gene.

### Statistics

The significance of differences between two groups was assessed by *t* test. The significance of differences between multiple groups was assessed by nonparametric approach (Dunnett’s test). The statistical analyses were performed using the JMP ver. 10.0 software (SAS Institute Inc., Cary, NC). *P* values less than 0.05 were considered significant.

## Results

### DPP4i inhibit the proliferation of effector CD4^+^ and CD8^+^ T cells

**Fig. 1:**
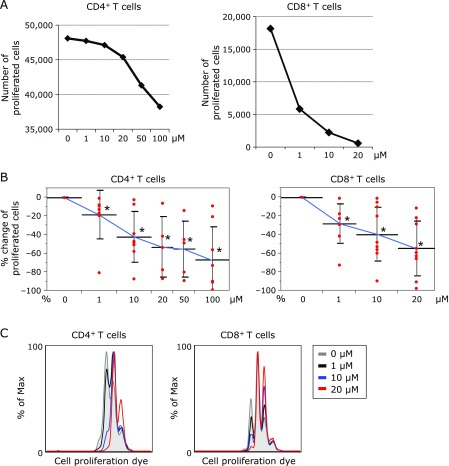
Adverse effect for the proliferation of naïve CD4^+^ and CD8^+^ T cell by DPP4 inhibitors. (A) Representative numbers of proliferated CD4^+^ T cells are shown. About 4 to 5 × 10^6^ cells were used. (B) The % changes of the rate of proliferated CD4^+^ and CD8^+^ T cells are plotted. The cell number at the culture condition without DPP4i is used as a reference. The data is expressed as mean ± SD. The experiments were repeated eight (0, 1, 10, and 100 µM) or six (20 and 50 µM) times. The significance of differences between multiple groups was assessed by nonparametric approach. *p<0.05. (C) Representative histogram of fluorescence intensity dyed by Violet Cell Proliferation Kits is shown. Gray line indicates the cultured cells under no DPP4i. Black line indicates the cultured cells under 1 µM of DPP4i. Blue line indicates the cultured cells under 10 µM of DPP4i. Red line indicates the cultured cells under 20 µM of DPP4i.

**Fig. 2:**
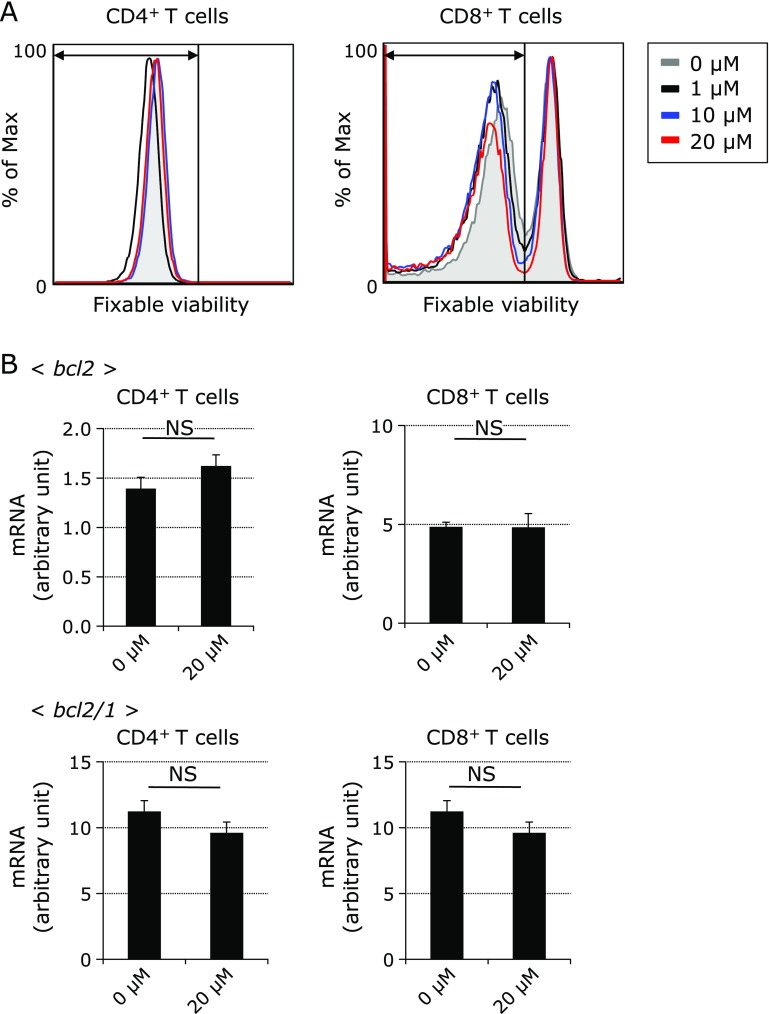
No harmful effect for the proliferation of naïve CD4^+^ and CD8^+^ T cell by DPP4 inhibitors. (A) Alive cells at the indicated culture conditions are shown as representative histogram of fluorescence intensity dyed by Fixable Viability Dye (FV). Alive cells are gated. Gray line indicates the cultured cells under no DPP4i. Black line indicates the cultured cells under 1 µM of DPP4i. Blue line indicates the cultured cells under 10 µM of DPP4i. Red line indicates the cultured cells under 20 µM of DPP4i. (B) The arbitral units of *bcl2* and *bcl2/1* expressions are shown as mean ± SD. NS indicates not significant.

**Fig. 3:**
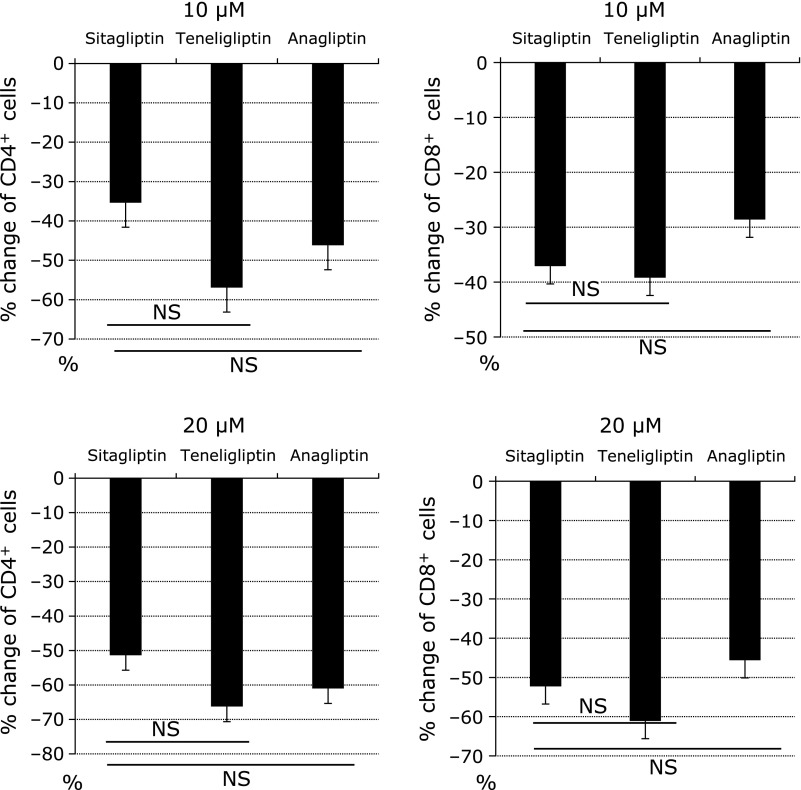
The class effect of cell proliferation assay. The % changes of the induction rate of proliferated CD4^+^ T cells and CD8^+^ T cells by administrating each DPP4i (10 and 20 µM) are indicated as bar graph. The data is expressed as mean ± SD. The experiments were repeated three times. The induction rate compared with sitagliptin is assessed by paired *t* test. NS indicates not significant.

The proliferation of CD4^+^ and CD8^+^ T cells was suppressed by adding DPP4i in a dose dependent manner (Fig. 1A and B). Assessment of the proliferative status was detected by the expression of Violet dye (CellTrace Violet Cell Proliferation Kit), which is used for *in vitro* and *in vivo* labeling of cells to trace multiple generations using dye dilution by flow cytometry. Representative histogram plots show the Cell Proliferation Dye fluorescence of CD4^+^CD3^+^ and CD8^+^CD3^+^ T cells stimulated by anti-CD3/CD28 for five days with four phase density (0, 1, 10 and 20 µM) of DPP4i (Fig. 1C). The greater part of CD4^+^ T cells stopped the proliferation or died by adding 50 and 100 µM of DPP4i. A lot of CD8^+^ T cells stopped the proliferation or died by adding equal to or more than 20 µM of DPP4i. To evaluate the cytotoxicity, we stained the culture cells with FV. The proportion of FV positive dying cells did not increase in the presence of DPP4i, as shown in the representative figures of flow cytometric analysis (0, 1, 10 and 20 µM) (Fig. 2A). Apoptosis associated genes including bcl2 and bcl2/**1 were also checked by real time PCR. We found that the expression levels of these genes were not significantly changed by administering DPP4i (20 µM) (Fig. 2B). We demonstrated these phenomena by adding 3 types of DPP4i we used (Fig. 3). Based on these results, DPP4i might have adverse effect for proliferation and activation of naïve CD4^+^ and CD8^+^ T cells.

### Phosphorylation of intracellular kinase is decreased in ERK, p38 MAPK and Akt signaling by DPP4i

**Fig. 4:**
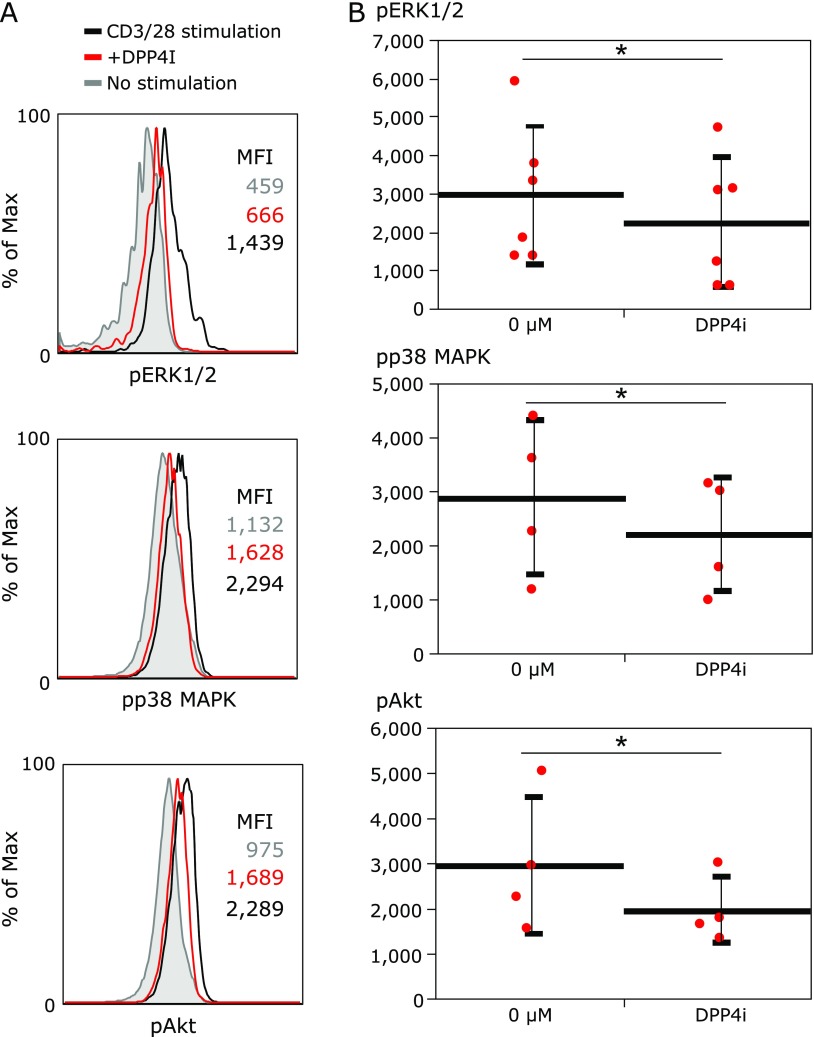
The modulation of extracellular-signal-regulated kinases (ERK) signaling pathway by DPP4 inhibitors. (A) The histogram analysis of fluorescence intensity of phospho extracellular-signal-regulated kinases (pERK) 1/2, phospho p38 mitogen-activated protein kinase (pp38 MAPK) and phospho Akt (pAkt) assesed by phosphor-flow analysis. Mean fluorescence intensity (MFI) is shown in the figure. Gray line and gray MFI indicates fluorescence intensity of pERK 1/2, pp38 MAPK and pAkt at the condition of no stimulation. Black line and black MFI indicates fluorescence intensity of pERK 1/2, pp38 MAPK and pAkt at the condition of stimulation with anti-CD3 and anti-CD28 antibody. Red line and red MFI indicates fluorescence intensity of pERK 1/2, pp38 MAPK and pAkt at the condition of stimulation with DPP4i (100 µM). (B) MFIs of each experiments are plotted. The data is expressed as mean ± SD. The change is assessed paired *t* test. The experiments were repeated four times (pp38 MAPK and pAkt) or six times (pERK1/2). *p<0.05.

The expression levels of the phosphorylation of pERK1/2, pp38 MAPK and pAkt decreased by adding DPP4i (Fig. 4). It was considered that the TCR signaling was attenuated in the presence of DPP4i.

### DPP4i don’t inhibit the T cell cytokine production

 To evaluate whether DPP4i inhibit the T cell cytokine production or not, we performed cytokine assay. As representative dot plots show, the percentage of cytokine producing from CD4^+^ T cells stimulated by PMA/IONO with sitagliptin (100 µM) are not different from those without DPP4i (Supplemental Fig. 1*). T cells maintained the function of the cytokine production after stimulated by PMA/IONO with DPP4i in the cytokine assay. We demonstrated this phenomenon by adding 3 types of DPP4i we used (Supplemental Fig. 2*). DPP4i did not inhibit cytokine production from CD4^+^ T cells.

### Physiological concentration of DPP4i

The information of the concentrations of sitagliptin, teneligliptin and anagliptin after oral administration to healthy Japanese volunteers and rats were provided by each pharmaceutical company. It was showed that when single dose of sitaligliptin (50 and 100 mg), teneligliptin (20 and 40 mg) and anagliptin (200 and 400 mg) were administered orally to healthy Japanese volunteers, the concentration of sitagliptin, teneligliptin and anagliptin in blood plasma can be calculated based on the following expressions; a maximum concentration of sitagliptin (*C*max) = 0.309 and 0.959 µM,^(13)^ teneligliptin (*C*max) = 0.18720 and 0.3824 µg/ml = 2.98 and 6.08 µM,^(14)^ and anagliptin (*C*max) = 1,040 and 3,330 ng/ml = 2.71 and 8.68 µM.^(15)^ The tissue concentration of each DPP4i after oral administration to rats were also shown by each manufacturer.^(13–15)^ Hence, it was validated that the range of the concentration of 1 to 100 µM which we used in the *ex vivo* experiment was physiological concentration after administration of each DPP4i.

## Discussion

In the current study, we demonstrated that DPP4i modulated TCR signaling and inhibited the proliferation of effector CD4^+^ T cells and CD8^+^ T cells in a dose dependent manner. We didn’t observe a kind of class effect among 3 types of DPP4i we used.

We hypothesized that pleiotropic effects of DPP4i might be derived from immunomodulatory effects of DPP4i via modulation of cell signaling at first. To evaluate the possibility, we performed *ex vivo* assays by using primarily human CD4^+^ T cells and CD8^+^ T cells.

The adverse effects for the proliferation of T cells were consistent with another study.^(11,16)^ It was revealed by phospho-flow that the downstream TCR signaling was attenuated in the presence of DPP4i. DPP4/CD26 is a co-stimulatory molecule on T cells by binding to caveolin-1 in antigen-presenting cells.^(7)^ In another study, inhibition of DPP-4/CD26 by alogliptin, one of DPP4i, suppressed Toll-like receptor 4-mediated ERK activation of antigen presenting cells.^(17)^ We also assumed that DPP4i associated blockade of CD26-caveolin-1 interaction and investigated the T cells stimulated by anti-CD3 and anti-CD28 antibodies in this study. Then, we effort to establish a possible mechanism of TCR modulation by studying the phosphorylation of ERK and MAPK. However, it remains unclear how inhibition of the catalytic site of co-stimulatory molecules not targeted could affect TCR signaling.

DPP4i did not inhibit cytokine production in this study, although numerous studies showed opposite with strong effects *in vitro* as well as *in vivo*.^(5,7,11,16)^ In the previous study, T cells, which were under blockade of CD26, demonstrated the decrease of cytokine production.^(7)^ On the other hands, the cytokine production from CD4^+^ T cells was not changed in the presence or absence of DPP4i when the naïve T cells were used in this study. DPP4i could not overwrite the very strong stimulation such as PMA/IONO, even if DPP4i interfered with TCR signaling. In addition, FACS is not best method to quantify cytokine production, as merely test the ability of cells to produce a cytokine, but not quantify. Further analyses should be considered to clarify this point.

It has also been demonstrated that at least some effects of DPP4i are mediated by off-target inhibition of other dipeptidyl peptidase of the DPP4 activity and structure homologue proteins.^(6,18)^ Thus, the observed effects might be mediated by other molecules, such as dipeptidyl peptidase 8 and 9, although the three DPP4i which we used had high selectivity for DPP4.^(13–15)^ We could not clarify which molecules were associate. Further study was needed in these point of view. 

Taken together, DPP4i modulated TCR signaling, which contributed to attenuate the proliferation of CD4^+^ T cells and CD8^+^ T cells.

Aside from their glucose-lowering action, DPP4i have several pleiotropic effects beyond the metabolism of incretin,^(1,2,19)^ which are also associated with potentially organ protective effects,^(20)^ cell survival signaling and extracellular matrix remodelings due to their diversity.^(21–23)^ Sitagliptin could inhibit the development of hepatic steatosis and reduced indomethacin-induced intestinal injury.^(24,25)^ DPP-4/CD26 is present in various tissues and cells including lymphocytes and monocytes as a transmembrane glycoprotein and is associated with immunoregulatory functions.^(5,6)^ DPP4i prolong islet graft survival in nonobese diabetic mice by modulating migration of pathogenic CD4^+^ T cells.^(26,27)^ Then, it is suggested that DPP4i might modulate autoimmunity by inhibiting pathogenic T cells.^(28)^ Furthermore, DPP4i decreased risk for rheumatoid arthritis and other autoimmune disease in patients with type 2 diabetes.^(12)^ These immunomodulatory effects might be associated with regulation of TCR signaling in T cell fate.

In type 2 diabetes, DPP4i are expected to have protective effect against chronic inflammation. Recent clinical trials showed that DPP4i did not increase the rate of major adverse cardiovascular events,^(29–32)^ and reduced the development and progression of diabetic nephropathy in patients with type 2 diabetes.^(29)^ Further studies would be needed to investigate cardiovascular benefits of DPP4i. One of interpretable mechanism in these phenomena might be immunomodulatory effects of DPP4i, which we demonstrated in the study. These immunomodulatory effects might contribute to control chronic inflammation in patients with type 2 diabetes.

There are several limitations in this study. First, the human PBMC was obtained from only a few Japanese healthy men; therefore, it is uncertain whether these findings can be generalized to other ethnic groups and the patients with type 2 diabetes. Second, because we performed *ex vivo* assays by using primarily human CD4^+^ T cells and CD8^+^ T cells by adding DPP4i in a dose dependent manner, the concentrations of each DPP4i was increased more than *in vivo* analysis. Third, there was a little difference between each DPP4i in a % changes of the proliferated CD4^+^ T cells and CD8^+^ T cells. The difference may be caused by the inhibitory action of each DPP4i which we could not evaluate in this study. Further studies are needed to confirm the difference of the proliferated CD4^+^ T cells CD8^+^ T cells by adding other DPP4i. Finally, this study is very preliminary in nature and does not go far enough to provide an in depth mechanistic explanation of the mode of action of DPP4i, although we might provide some potentially useful observations with regards to immunomodulatory effects.

## Conclusion

In conclusion, DPP4i affect TCR signaling and have adverse effects for the proliferation and activation of human naïve T cells.
