# Supplementary material for: Dipeptidyl peptidase-4 inhibitors have adverse effects for the proliferation of human T cells
Source: J Clin Biochem Nutr. 2018 Apr 3;63(2):106–12. doi: 10.3164/jcbn.17-64 (PMC6160731; doi:10.3164/jcbn.17-64)
Supplement: Supplemental Fig. 1 [file jcbn17-64sf01.pdf]

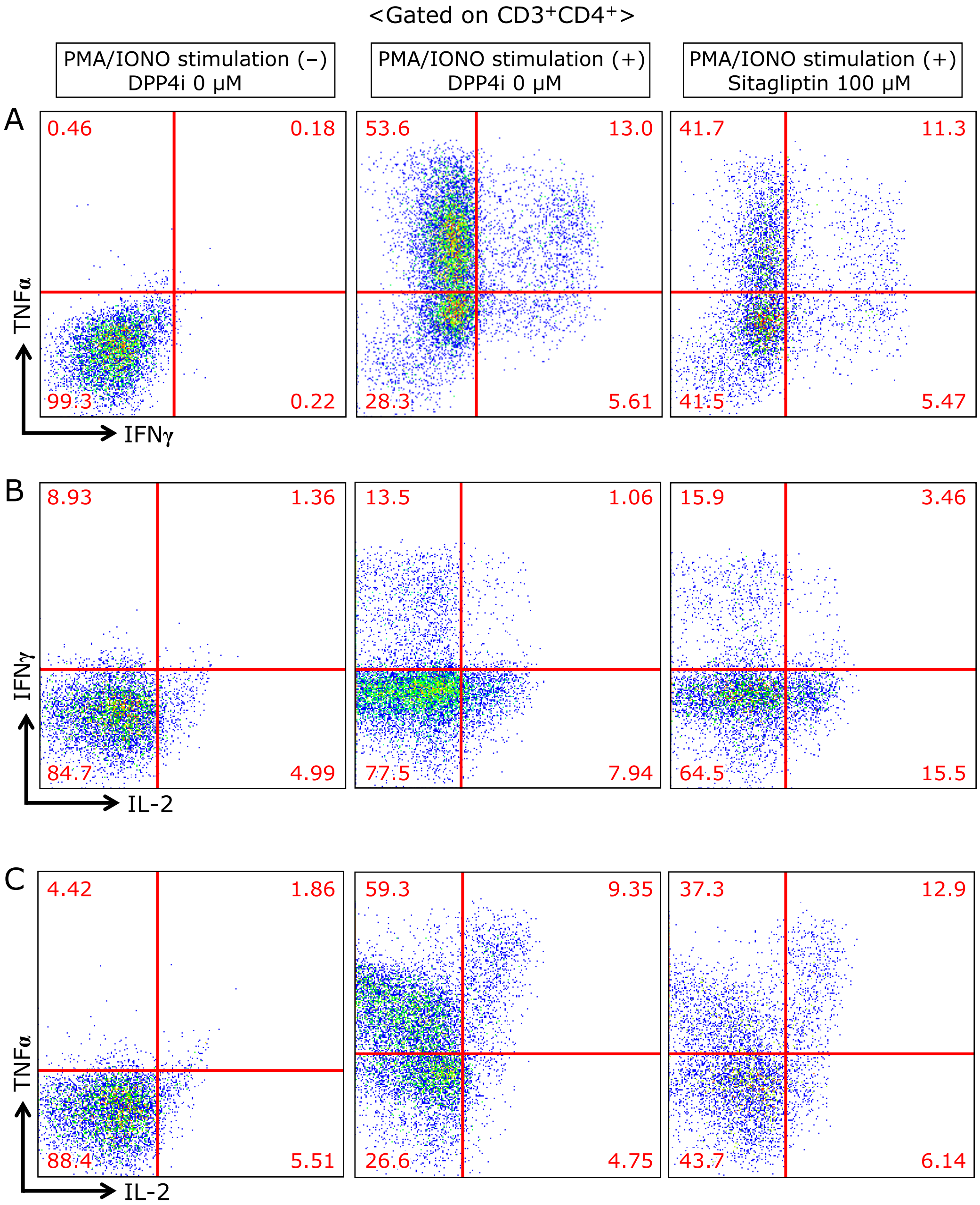

Supplemental Fig. 1. No harmful effect for the cytokine production of CD4+ T cell by DPP4i. To investigate whether DPP4i inhibited the T cell cytokine production, we performed cytokine assays, using PMA and IONO to stimulate peripheral blood mononuclear cells. Representative pseudo color dot plots IFNγ and TNFα producing cells (A), IL2 and IFNγ producing cells (B) and IL2 and TNFα producing cells (C) are shown. Numbers indicate the proportion of quadruple fractions.
