# Supplementary material for: Dipeptidyl peptidase-4 inhibitors have adverse effects for the proliferation of human T cells
Source: J Clin Biochem Nutr. 2018 Apr 3;63(2):106–12. doi: 10.3164/jcbn.17-64 (PMC6160731; doi:10.3164/jcbn.17-64)
Supplement: Supplemental Fig. 2 [file jcbn17-64sf02.pdf]

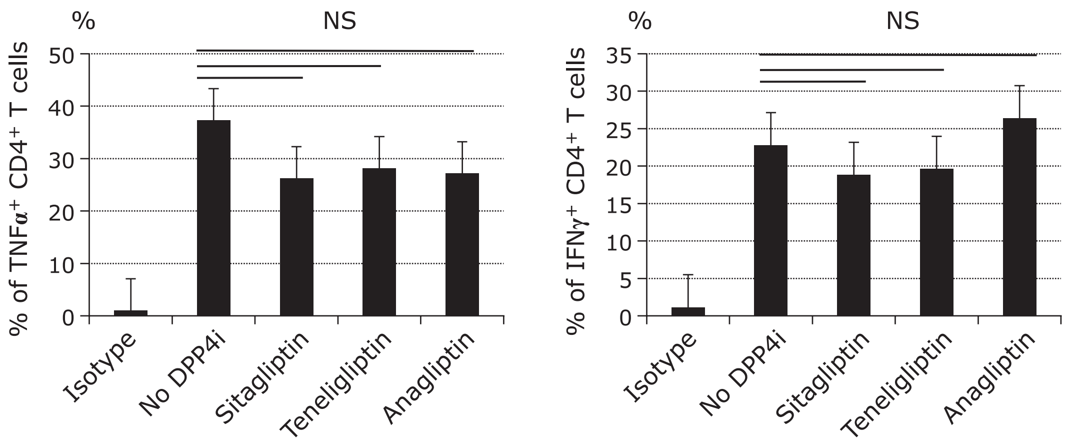

Supplemental Fig. 2. The class effect of cytokine assay. The bar graphs show the % of TNFα and IFNγ producing cells stimulated by PMA and IONO with or without each DPP4i (sitagliptin 100 μM, teneligliptin 1 μM, and anagliptin 1 μM). The data is expressed as mean ± SD. The experiments were repeated two times. The rate of TNFα and IFNγ producing compared with the control (stimulated by PMA and IONO without DPP4i) is assessed by paired *t* test. NS indicates not significant.
